# Comprehensive In Vitro Safety Assessment of *Acorus calamus* Rhizome Oil Using OECD-Compliant New Approach Methods: Classification as a GHS Category 1B Sensitiser and Category 2 Irritant

**DOI:** 10.3390/toxics13121006

**Published:** 2025-11-21

**Authors:** Karishma R. Desai, Jay R. Ranade, Rajendra M. Nagane, Manish V. Patel, Abhay D. Deshpande, Clive S. Roper, Gireesh Babu Kantli

**Affiliations:** 1 Department of Toxicology (Mutagenicity), Jai Research Foundation, Valsad 396105, Gujarat, India; jay.ranade@jrfonline.com (J.R.R.); rajendra.nagane@jrfonline.com (R.M.N.); patelmv@jrfonline.com (M.V.P.); abhay.deshpande@jrfonline.com (A.D.D.); 2 Roper Toxicology Consulting Limited, Edinburgh EH3 6AD, UK; clive@ropertcl.com; 3 Department of Life Sciences, Parul Institute of Applied Sciences, Parul University, Vadodata 391760, Gujarat, India

**Keywords:** *A. calamus*, rhizome oil, skin irritation, skin corrosion, skin sensitisation, non-animal methods, new approach methodologies (NAMs)

## Abstract

**Background**: *Acorus calamus* (sweet flag) is widely used in traditional medicine, yet its dermal safety profile remains insufficiently defined under modern regulatory standards. **Objective**: To comprehensively evaluate the skin irritation, corrosion, and sensitisation potential of *A. calamus* rhizome oil using new approach methodologies’ (NAMs) test batteries under GLP conditions. **Results**: The *A. calamus* rhizome oil was predicted as a Category 2 skin irritant, non-corrosive and GHS Category 1B skin sensitiser. Chemical analysis revealed β-asarone as the major constituent (~40.75%). The reconstructed human epidermis models established reversible irritation without corrosion. Mechanistic concordance across the Direct Peptide Reactivity Assay, KeratinoSens™, and Human Cell Line Activation Test showed activation of the three key events of the skin sensitisation adverse outcome pathway. Using the “2-out-of-3” Defined Approach with the KE 3/1 sequential strategy allowed for hazard classification into GHS Category 1B. Quantitative risk modelling using SARA-ICE models and SCCS parameters yielded conservative safe-use concentrations ranging from 0.13 to 0.78% (*w*/*w*) for leave-on products and up to 7.46% (*w*/*w*) for rinse-off formulations. **Conclusions**: The combined evidence from the NAM-based assays showed that *A. calamus* rhizome oil is a moderate sensitiser and irritant but not corrosive, providing critical data for risk assessment and regulatory decision-making, which was previously unknown. The SARA-ICE PoD-derived safe-use concentrations provide guidance for cosmetic formulators to ensure consumer safety, particularly in leave-on applications such as face and hand creams, where sensitisation risk is highest. This study demonstrates the utility of NAMs for botanical safety assessment and regulatory decision-making.

## 1. Introduction

*Acorus calamus*, commonly known as calamus, sweet flag, or Pillai Marunthu, is widely recognised for its diverse medicinal properties, attributed primarily to the oil extracted from its rhizome [1]. This plant has a vast record of herbal uses [2]. The oil extracted from the rhizome has been traditionally used to treat diseases such as mental disorders, epilepsy, dysentery, chronic diarrhoea, abdominal tumours, fever, kidney and liver issues, and rheumatism [3,4]. Additionally, the mature leaves of *A. calamus* possess anthelmintic, antifungal, insecticidal, and antibacterial properties, further emphasising its therapeutic significance [5,6,7]. Beyond its medicinal uses, rhizome oil finds applications in the pharmaceutical industry [8]. Moreover, lectins in the rhizomes exhibit mitogenic activity [9]. *A. calamus* is also taken for a range of health conditions such as oral and throat diseases, epilepsy, bronchitis, fevers, delirium, tumours, hysteria, excessive thirst, memory impairment, rat bites, earworms, general fatigue, tooth pain, chest and kidney discomfort, asthma, diarrhoea, dysentery, flatulence, dyspepsia, chronic ulcers, and rheumatism [10]. It is also used as a remedy for snakebites and remittent fever [11]. Inhalation of the oil may alleviate low-grade fever and dyspepsia and enhance vocal clarity. Traditional use of *A. calamus* for colic and diarrhoea is supported by its calcium channel blocking activity demonstrated in rabbit jejunum [12,13].

Plant-based materials, despite their therapeutic benefits, may lead to adverse dermal reactions such as skin irritation, sensitisation, phototoxicity, and allergic responses [14]. Herbal ingredients in cosmetics, pharmaceutical formulations, and over-the-counter products are often associated with allergic contact dermatitis, a significant dermatological condition caused by chemicals interacting with living cells [15,16]. Results of published studies show that indeed, some herbs have toxic effects, with major hepatotoxic herbs being *Cimicifuga racemosa*, *Larrea tridentata*, *Teucrium chamaedrys*, *Scutellaria lateriflora*, and *Scutellaria baicalensis*, etc. [17,18]. Different herbal medicines also affect the heart, which comprises drugs obtained from plants such as *Digitalis purpurea* [19]. Several common plants used in herbal medicines have probable neurotoxic effects, e.g., *Papaver somniferum* (opium) [20]. This raises concerns about the potential toxic effects from both short-term and long-term use of these plants. Therefore, the evaluation of the toxicological outcomes of any herbal extract proposed to be used in humans is of paramount significance. Phytochemical characterisation, comprising the plant source, data on contamination, adulteration, and hazardous residues, is the critical issue in the safety assessment of plant materials in personal care products. The Defined Approach (DA), combining the Direct Peptide Reactivity Assay (DPRA), KeratinoSens™, and the human Cell Line Activation Test (h-CLAT), has been applied to a broad set of 181 regulatory-interest substances, including complex or otherwise challenging chemistries, to produce hazard and potency predictions suitable for downstream risk assessment and regulatory consideration, and highlights practical limits for mixtures and poorly soluble materials [21]. These authors recommended that DA-derived outcomes for multi-constituent botanical extracts should be interpreted alongside robust compositional characterisation and exposure information.

Skin irritation and skin sensitisation are critical aspects for the evaluation of the materials exposed to the skin. Allergic contact dermatitis, affecting an estimated 15–20.1% of the general population, is a prevalent condition, with cosmetics being the second leading cause of contact allergies due to the fragrances and preservatives they contain [22]. Given the widespread use of these products, avoiding exposure is challenging. Furthermore, many topically applied botanical products contain compounds capable of inducing contact dermatitis [23,24], necessitating systematic toxicological assessments. The skin toxicity is simplest to identify as the reaction is instantaneously noticed.

Plant materials or extracts comprise several adverse dermal effects, including skin irritation, skin sensitisation, phototoxicity, and immediate-type allergies. The increasing reliance on herbal products underscores the need for comprehensive safety and efficacy data [25]. Photosensitisation dermatitis is a toxic skin reaction triggered by exposure to sunlight when a photosensitiser is present in the body, leading to sunburn-like reactions in non-pigmented skin areas. Plant materials from *Ficus carica*, *Heracleum mantegazzianum*, Tetradymia species, Hypericum species, and *Lantana camara*, have been proven to trigger photosensitisation reactions [26]. Food materials from some plants also show skin irritation, e.g., *Pastinaca sativa* (parsnip) and *Agaricus bisporus* (mushrooms) [27].

Nonetheless, despite the number of ethnobotanical uses and reported bioactivities, the toxicological profile of *A. calamus* is heterogeneous and chemotype-dependent [28], and its toxicological profile remains insufficiently studied [29]. The pharmacological and toxicological impacts of these plant materials are reviewed in a publication that suggests the overall toxicity profile of the extract of *A. calamus* has demonstrated the cardiotoxic, hepatotoxic, reproductive toxic, mutagenic, and carcinogenic potential for propenyl asarone isomers [30].

Regulatory bodies and expert committees have therefore restricted or advised limits on the use of calamus/asarone-containing preparations: the United States Food and Drug Administration lists *A. calamus* among substances disallowed for direct addition or use as food [31]. European scientific reviews have concluded that β-asarone is carcinogenic in rodents and should be minimised in flavouring/food uses and fragrance-industry guidance (IFRA) sets conservative maximum levels for cis/trans-asarone in finished consumer products, with reported guidance for finished product concentrations being ≈100 ppm/0.01% [32]. These toxicological and regulatory data make compositional analysis (β-asarone content and chemotype identification) a critical component of any safety assessment of *calamus* rhizome oils [33]. The ban on *A. calamus* was centred on the carcinogenic effects observed in laboratory animals exposed to sweet flag extracts of the diploid variety, containing higher quantities of β-asarone [34]. Based on these developments, limits for β-asarone content in non-alcoholic and alcoholic drinks were set by the European Council to 0.1 mg/kg and 1 mg/kg, respectively [35,36]. Still, very limited or insufficient information is available to ascertain the toxic dose of *A. calamus* oil.

Although several studies conducted using the essential oils obtained from rhizomes of *A. calamus* have demonstrated numerous pharmacologic actions, very few or rare scientific studies have been published with recently innovated advanced technologies and models to prove these oils’ safety for different endpoints, such as their genotoxicity, skin sensitisation, or skin irritation effects. *A. calamus* was chosen for reasons directly relevant to risk management for skin sensitisation including the following: (1) its rhizome oil features in traditional remedies and contemporary leave-on and rinse-off personal-care formats, creating broad potential for consumer exposure [37,38]; (2) β-asarone is a prominent constituent with historical toxicological flags [39], making a transparent, point-of-departure (PoD)-based evaluation especially important; (3) the asarone isomer content varies by plant chemotype, geography [40], increasing uncertainty in extrapolating safety across supply chains; and (4) despite its traditional ethnomedicinal use, no single study that integrates a fully OECD-compliant battery with quantitative risk translation to a product-type specific maximum in use concentrations was found. Addressing these gaps for *A. calamus*, therefore, offers both ingredient-specific clarity and a general template for botanical oils with similar data deficiencies.

This prompted the current investigation to evaluate the rhizome extract for skin safety evaluations using new approach methodologies (NAMs), involving skin irritation, skin corrosion, and skin sensitisation assays. Modern regulatory practice and the adverse outcome pathway (AOP) for skin sensitisation emphasise mechanism-based, animal-free approaches that map to the key events of the sensitisation cascade with three key events (KEs); KE1, covalent protein binding (haptenation), assessed by the Direct Peptide Reactivity Assay (DPRA; OECD Test Guideline No. 442D); KE2, keratinocyte activation via Nrf2-ARE signalling, measured by the KeratinoSens™ assay (OECD Test Guideline No. 442D); and KE3, dendritic-cell activation, characterised by the human Cell Line Activation Test (h-CLAT; OECD Test Guideline No. 442E). Together, these assays form the foundation of DAs and Integrated Approaches to Testing and Assessment (IATA) that enable hazard identification and potency categorisation without reliance on animal models such as the Local Lymph Node Assay (LLNA) or Guinea Pig Maximisation Test (GPMT). Similarly, the use of reconstructed human epidermis models for assessing skin irritation and corrosion provides validated, regulatory-accepted tools for evaluating dermal toxicity. Collectively, these OECD-validated NAMs now provide a clear, mechanistically proven pathway not only for the classification of skin sensitisers, irritants, and corrosive agents, supporting both hazard assessment but also the quantitative next-generation risk management (NGRA) of cosmetic and botanical ingredients. Accordingly, the present study provides the first integrated, NAM-based toxicological profile for *A. calamus* rhizome oil, addressing a critical data gap and establishing its dermal hazard potential within a modern regulatory context.

### Test Guidelines and GLP Compliance

All experiments were conducted following the relevant OECD Test Guidelines (TGs). Skin sensitisation was determined using the skin sensitisation DA as highlighted in OECD TG No. 497 [41] following performance of OECD TG No. 442C for peptide reactivity using the DPRA [42], OECD TG No. 442D for keratinocyte activation using the KeratinoSens™ assay [43] and OECD TG No. 442E for dendritic cell activation using the hCLAT assay [44]. Skin irritation was assessed using the SkinEthic reconstructed human epithelium (RhE) model following OECD TG No. 439 [45]. Skin corrosion was assessed using the RhE model following OECD TG No. 431 [46]. All studies made a formal claim of GLP compliance.

## 2. Materials and Methods

### 2.1. Plant Material and Extraction of Rhizome Oil

The *A. calamus* rhizomes were collected from the Pampore, Pulwama District, Jammu and Kashmir, India (Geographical coordinates, 33.92882 74.90524 34.11682 75.09093). Post-collection, the rhizomes were air-dried and finely ground using an auto mixer (Figure S1). Dried rhizome powder was mixed with distilled water and subjected to hydro-distillation using a Clevenger-type apparatus, Borosil, Mumbai, India [47] (Figure S2). The mixture was heated to 100–120 °C for 4 h, facilitating efficient extraction of the oil.

The oil was separated from the aqueous solution after no further condensing oil was visible, and after extraction, the resultant essential oil was stored at 4 °C for subsequent analysis and experimental use.

### 2.2. Chemicals and Reagents

Unless otherwise specified, all chemicals and materials adhered to the requirements described in the OECD test guidelines (TG Nos. 442C, 442D, 442E, 439, and 431). The following chemicals and reagents were used: Dulbecco’s Phosphate-Buffered Saline (DPBS), Foetal Bovine Serum (FBS), Trypsin-EDTA (0.05%), Dulbecco’s Modified Eagle Medium with Glutamax, and Geneticin; all were sourced from Gibco, Grand Island, NY, USA. Maintenance Medium and Growth Medium were obtained from SkinEthic Laboratories, Lyon Cedex 7, France. Dimethyl sulfoxide (DMSO), sodium hydroxide, isopropanol, and nickel sulphate were purchased from Qualigens, Mumbai, India. Trans-cinnamaldehyde, 2,4-dinitrochlorobenzene (DNCB), and 3-(4,5-dimethylthiazol-2-yl)-2,5-diphenyltetrazolium bromide (MTT) were sourced from Sigma-Aldrich, St. Louise, MO, USA. IgG1 and anti-CD54 was obtained from BD Pharmingen, Franklin Lakes, NJ, USA, while anti-CD86 was obtained from Dako, Carpinteria, CA, USA. Propidium iodide was purchased from Sigma-Aldrich, St. Louise, MO, USA. Penicillin-streptomycin was obtained from Thermo Fisher Scientific, Darmstadt, Germany. Passive lysis buffer was obtained from Promega, Madison, WI, USA.

### 2.3. Determination and Quantification of α-, β-, and γ-Asarone

#### 2.3.1. Chemicals and Reference Standards

Certified analytical reference standards of α-asarone (TCI, Haryana, India; purity = 99.7%), β-asarone (Phytolab, Vestenbergsgreuth, Germany; purity = 96.41%), and γ-asarone (Biorbyt, Cambridge, UK; purity = 99.35%) were used. Individual stock solutions (1000 mg/L) were prepared in HPLC-grade methanol by accurately weighing α-asarone (1.004 mg), β-asarone (1.038 mg), and γ-asarone (1.007 mg) into separate 1 mL volumetric flasks. Ten-fold dilutions in methanol yielded 100 mg/L working standards for calibration, retention time confirmation, and mass-spectral identification.

#### 2.3.2. Sample Preparation for HPLC Analysis

A weighed quantity of 20 mg of hydro-distilled *A. calamus* oil was made up to 1 mL, to prepare the stock solution (20,000 mg/L) for HPLC analysis. This stock was diluted 10 times in methanol to obtain 2000 mg/L. Aliquots were further diluted to prepare analyte-specific test concentrations: α-asarone (100 mg/L); β-asarone (1250 mg/L); and γ-asarone (1000 mg/L). The final test solutions were injected directly into HPLC and LC-MS/MS systems without additional treatment.

#### 2.3.3. HPLC Quantification

Quantitative analysis was performed on a i-series LC-2050C system equipped with a UV detector, sourced from Shimadzu, Kyoto, Japan. Separation was achieved on an Agilent Eclipse C18 column (150 mm × 4.6 mm, 3.5 µm), sourced from Bengaluru, Karnataka, India, maintained at 40 °C. The mobile phase comprised formic acid in methanol (0.1%)–water (60:40, *v*/*v*) delivered isocratically at 0.7 mL/min. The autosampler was maintained at 15 °C, the injection volume was 5 µL, and UV detection was set at 254 nm.

#### 2.3.4. LC-MS/MS Confirmation

Analyte identity was confirmed on a 8050 LC-MS/MS coupled to a Nexera-X2 UHPLC sourced from Shimadzu, Kyoto, Japan. Chromatographic separation employed a Union column with methanol ammonium formate (5 mM) in water (85:15, *v*/*v*) at 0.6 mL/min. Injections (2 µL) were analysed with the autosampler at 6 °C and the column at 40 °C. Mass spectrometry was performed in positive electrospray ionisation mode using a Q1 scan range of 270–300 *m*/*z*. Interface, desolvation line, and heat-block temperatures were 300 °C, 250 °C, and 400 °C, respectively.

#### 2.3.5. Quantification and Data Analysis

Analyte concentrations in the test samples were calculated based on the area responses of standards (AS) and samples (AT), reference standard concentration (RC), volume (mL) of reference standard taken from stock solution (RT), final volume (mL) of working solution of reference standard (RV), concentration of sample stock solution (SC), final volume (mL) of working solution of sample solution (ST), and volume (mL) of stock solution of sample taken, according to the following equation:

$$\%\mathrm{Content}=\;\frac{\mathrm{AT}}{\mathrm{AS}}\times \left(\mathrm{RC}\right)\times \frac{\mathrm{RT}}{\mathrm{RV}}\times \left(\mathrm{SC}\right)\times \frac{\mathrm{ST}}{\mathrm{SV}}\times 1000$$

#### 2.3.6. Solubility Test

The solubility of *A. calamus* rhizome oil in DMSO was checked and was found to be appropriate for use. Dose formulations for the hCLAT and KeratinoSens^TM^ assays were prepared in DMSO and then diluted in relevant media. For the DPRA test, the rhizome oil was dissolved in acetonitrile. The highest concentrations of the extracted oil used for testing were 500 mg/mL (h-CLAT), 40 mg/mL (KeratinoSens™), and 20 mg/mL (DPRA), with the DMSO concentration limited to no greater than 0.2–1% (*v*/*v*). For the skin Irritation and skin corrosion test, hydro-distilled *A. calamus* rhizome extract was used without any further dilution.

#### 2.3.7. In Vitro Skin Irritation and Corrosion Tests

To evaluate the skin irritation and corrosion potential of *A. calamus* rhizome oil, in vitro tests were conducted using the SkinEthic™ RhE model. This model replicates the multi-layered, highly differentiated, and stratified epidermis structure of human skin. The skin irritation test identifies irritants (UN GHS Category 2) and non-irritants (UN GHS No Category), while the skin corrosion test distinguishes corrosive substances (UN GHS Category 1) from non-corrosive substances. Both tests rely on measuring cell viability through enzymatic conversion of the vital dye, MTT, to formazan, which is quantitatively measured to assess tissue damage.

#### 2.3.8. Skin Irritation Test

Skin irritation was assessed using the SkinEthic™ reconstructed human epidermis (RhE) model in accordance with OECD TG No. 439. Tissues were pre-incubated for at least 2 h at 37 ± 1 °C, 5% CO_2_. *A. calamus* rhizome oil was applied directly onto tissues (16 µL/0.5 cm^2^) with concurrent negative (DPBS) and positive (5% SDS) controls; freeze-killed tissues were used to correct for non-specific MTT reduction. After the prescribed exposure and post-incubation intervals, the tissues were processed for an MTT viability assessment, and absorbance was read at 570 nm. Mean tissue viability (%) relative to the negative control was used to determine irritancy according to the OECD TG No. 439 acceptance criteria. Detailed experimental conditions are provided in Supplementary Materials (Tests S1–S3).

#### 2.3.9. Skin Corrosion Test

Skin corrosion was evaluated using the SkinEthic™ RhE model in accordance with OECD Test Guideline No. 431. Tissues were pre-incubated for at least 2 h at 37 ± 1 °C, 5% CO_2_. *A. calamus* rhizome oil was applied directly onto tissues (40 µL/0.5 cm^2^) for 3 min and 60 min alongside negative (sterile water) and positive (8 N KOH) controls. Freeze-killed tissues were included to correct for non-specific MTT reductions. After exposure, tissues were rinsed, incubated with MTT, and formazan extraction and absorbance measurements at 570 nm were performed as described for the irritation assay. Tissue viability relative to negative controls determined corrosion potential according to the OECD TG No. 431 criteria. The detailed experimental conditions are provided in Supplementary Materials (Texts S1–S3).

#### 2.3.10. The Direct Peptide Reactivity Assay (DPRA)

The Direct Peptide Reactivity Assay (DPRA) addresses the first key event (KE1) of the skin sensitisation AOP, assessing the covalent binding of electrophilic test items to nucleophilic residues in model peptides containing cysteine or lysine. The method was conducted in accordance with OECD TG No. 442C and DB-ALM Protocol 154. Synthetic cysteine and lysine peptides (0.667 mM) were incubated individually with *A. calamus* rhizome oil (20 mg/mL in acetonitrile) at defined 1:10 and 1:50 molar ratios, respectively, for 24 ± 2 h at room temperature in dark. Cinnamaldehyde (100 mM) served as the positive control, and acetonitrile as the vehicle control. Following incubation, peptide depletion was quantified by HPLC-UV at 220 nm using a reverse-phase C-18 column and expressed as percentage loss relative to control samples. The assay’s acceptance criteria, including reference control stability and linearity, were met for all runs. Classification of sensitising potential was based on the mean percent depletion of cysteine and lysine peptides, using the prediction model threshold of 6.38% to discriminate sensitisers from non-sensitisers. Reactivity classes (low, moderate, and high) were assigned according to OECD-defined depletion ranges for integration into the DA (OECD TG No. 497). The detailed procedure for the assay is described in Supplementary Materials (Texts S1–S3).

#### 2.3.11. KeratinoSens™ Assay

To further assess the skin sensitisation potential of *A. calamus* rhizome oil, the KeratinoSens™ assay was conducted. This assay evaluates keratinocyte activation, the second key event in the AOP for skin sensitisation, by measuring the Keap1-Nrf2-ARE-mediated activation of antioxidant response element (ARE)-dependent genes. The assay utilises the stably transfected HaCaT cell line, KeratinoSens^TM^ (acCELLerate GmbH, Osterfeldstraße 12–14, 22529 Hamburg, Germany), and quantifies luciferase activity as an indicator of Nrf2 pathway activation. A significant increase in luciferase activity, exceeding predefined thresholds and occurring at non-cytotoxic concentrations, was considered indicative of a positive sensitisation response.

The cells were seeded in 96-well plates at a density of 10,000 cells/well, one day prior to exposure. Plates were incubated at 37 ± 1 °C in a 5 ± 1% CO_2_ atmosphere for 24 h. *A. calamus* rhizome oil was tested at concentrations ranging from 0.20 to 400 µg/mL in DMSO. Trans-cinnamaldehyde was used as the positive control, and cells with DMSO were used as the negative control. One well with untreated cells was kept as a blank. Four plates were seeded for testing; three 96-well white assay plates for the luciferase assay, and one 96-well flat-bottom transparent plate for the cytotoxicity (MTT) assay was prepared. Following treatment, plates were incubated for 48 ± 2 h at 37 ± 1 °C, in a 5 ± 1% CO_2_ atmosphere. After incubation, cytotoxicity was assessed using the MTT assay. The MTT solution (5 mg/mL in DPBS) was mixed in DMEM containing FBS (1%, *v*/*v*). After the addition of MTT, plates were incubated for 4 h before adding isopropanol (50 µL) to dissolve the formazan crystals. Plates were shaken for 30 min and absorbance was measured at 570 nm using a Synergy HT microplate reader (BioTek Instruments, Winooski, VT, USA).

The cytotoxicity test was performed to make sure the observed luciferase induction was not due to adverse effects resulting from cell death.

For the luminescence measurement, the medium was removed, and the cells were washed once with DPBS. Passive lysis buffer was added to each well and plates were incubated for 20 min at room temperature. The luciferase substrate was added and luminescence was measured.

The assay evaluates the luciferase induction at various concentrations of the *A. calamus* rhizome oil. Results exceeding the predefined thresholds of EC_1.5_ (i.e., I_max_ ≥ 1.5-fold compared to solvent control) were considered positive for skin sensitisation response.

#### 2.3.12. Human Cell Line Activation Test (h-CLAT)

The skin sensitisation potential of *A. calamus* rhizome oil was evaluated using the h-CLAT test method. This assay, based on the THP-1 cell line (ATCC: TIB-202), is a surrogate model for dendritic cells (DCs) and is described in OECD TG No. 442E for assessing skin sensitisers. The method quantifies the upregulation of CD86/CD54 on the THP-1 cell line (mimicking the activation of DCs), which correlates with the substance being a sensitiser. The h-CLAT method is widely adopted by regulatory authorities for differentiating between skin sensitisers and non-sensitisers.

THP-1 cells were cultured in RPMI-1640 medium supplemented with (FBS, 10% *v*/*v*), 2-mercaptoethanol (0.05 mM), and penicillin (100 U/mL)–streptomycin (100 µg/mL). Before the experiments, cells were tested for their ability to express CD86 and CD54 surface markers in response to the positive controls (reactivity check).

In the DRF experiments, the cells were exposed to ten (1.75–1000 µg/mL), 2-fold serial dilutions of *A. calamus* rhizome oil in DMSO across two independent experiments. The CV_75_ value (the concentration resulting in 75% cell viability) was determined using propidium iodide staining and flow cytometry. Working solutions of the test items and controls were prepared in a 1:1 (*v*/*v*) ratio with cell suspensions in 96-well flat-bottom plates.

Based on the results of the dose range-finding assay, eight concentrations were selected at 1.2-fold dilutions for assessing CD86 and CD54 expression. The highest dose corresponded to CV_75_ × 1.2-fold. Cells were exposed to the final concentration of 17–61 µg/mL of *A. calamus* rhizome oil concentrations, with DNCB included as the positive control at a final concentration of 8 µg/mL. Controls for the medium (FBS in RPMI 1640; 10%, *v*/*v*) and solvent (DMSO at 0.2%, *v*/*v*) were included in both the dose range finding and CD86/CD54 assays. Incubations were conducted for 24 h at 37 °C in a 5% CO_2_ atmosphere.

After incubation, cells were washed with a staining buffer PBS containing BSA (0.1%, *w*/*v*), followed by the FcR blocking, followed by staining with anti-human CD86-FITC and anti-human CD54-FITC and mouse IgG1 antibodies. After washing, the cells were stained with propidium iodide (0.625 µg/mL) to distinguish between live and dead cells. The samples were analysed using flow cytometry (FACS Lyric^TM^, BD, Franklin Lakes, NJ, USA) and the relative fluorescence intensity (RFI) of CD86 and CD54 was calculated. The *A. calamus* rhizome oil was classified as a skin sensitiser if the RFI exceeded the established thresholds (RFI ≥ 150 for CD86 and RFI ≥ 200 for CD54). An increase in these markers indicates the activation of dendritic cell-like responses, suggesting potential skin sensitisation.

## 3. Results

### 3.1. The A. calamus Rhizome Oil Is Dominated by Beta-Asarone

**Figure 1:**
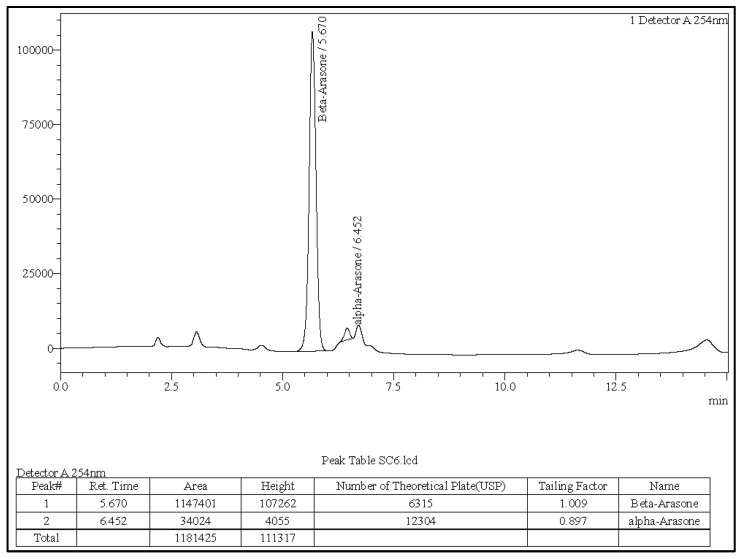
*A. calamus* rhizome oil sample solution.

The α-asarone, β-asarone, and γ-asarone were baseline separated with the expected retention times. Peak morphology demonstrated optimal symmetry (tailing factor < 1.1), and column performance was confirmed by theoretical plate counts exceeding 5800. Analysis of the *A. calamus* rhizome oil produced distinct peaks corresponding to α-asarone and β-asarone, while γ-asarone was not detected under these conditions (Figure 1). LC-MS/MS analysis verified molecular ions for all three isomers within the anticipated mass range (*m*/*z* 270–300), confirming clear identification of each asarone isomer.

Retention times of the detected analytes matched those of the reference standards and LC-MS/MS spectra confirmed their molecular signatures. Representative chromatograms for the α, β, and γ reference standards are provided in Figures S3–S5.

### 3.2. Quantitative Determination of Asarone Isomers

Quantification was based on peak area ratios of the sample to the corresponding standards. Calculated concentrations of each analyte in the *A. calamus* rhizome oil are summarised in Table 1.

The rhizome oil contained a predominant proportion of β-asarone (40.75%) and a moderate amount of α-asarone (4.16%). γ-asarone was below the detection limit of the method. Concordance of retention times and LC–MS/MS spectra between standards and sample peaks ensured the accuracy of analyte identification and quantification.

### 3.3. The Skin Irritation Test Results Show That A. calamus Rhizome Oil Is an Irritant

The tissue viability for *A. calamus* rhizome oil and the positive control was measured as a percentage of the negative control and NS_MTT_ was calculated (Table 2). Tissues treated with DPBS (negative control) resulted in high viability across replicates. The mean viability of tissues treated with *A. calamus* rhizome oil was 23.5% (standard deviation (SD) = 1.50%, coefficient of variation (CV) = 6.39%) compared to the negative control. Tissues treated with positive control (5% SDS) showed a mean viability of 1.5% (SD = 0.058%, CV = 3.87%), indicative of suitable assay performance. The NS_MTT_ values for the negative control were negligible, confirming no interference with the MTT assay. The NS_MTT_ values for the *A. calamus* rhizome oil were negative, with a mean value of -11.2%, which demonstrated that the rhizome oil did not cause a non-specific reduction in MTT. The individual tissue replicates’ viability for negative, positive, and *A. calamus*-treated rhizome oil is provided in Figure S6.

### 3.4. The Skin Corrosion Test Identified A. calamus Rhizome Oil as Non-Corrosive

The tissue viability of *A. calamus* rhizome oil and the positive control was measured as a percentage of the negative control, with NS_MTT_ assessed to confirm the accuracy of the results. After 3 min of exposure (Table 3), *A. calamus* rhizome oil-treated tissues showed a mean viability of 92.72% (SD = 2.13%, CV = 2.30%), compared to the negative control. These results showed minimal impact on the tissue viability within the 3 min exposure period. The NS_MTT_ for *A. calamus* rhizome oil was negligible, with a mean value of −0.7%, confirming that the *A. calamus* rhizome oil itself did not interfere with MTT. After 60 min of exposure (Table 3), *A. calamus* rhizome oil-treated tissues retained a mean viability of 91.17% (SD = 1.49%, CV = 1.63%). The positive control (8N KOH) reduced tissue viability to 0.17% (SD = 0.03%, CV = 17.65%), confirming the assay’s performance. NS_MTT_ for *A. calamus* rhizome oil during the 60 min exposure was also negligible, with a mean value of 0.1%. The negative control (distilled water) showed high tissue viability for both exposure times, while the positive control reduced viability to nearly zero, confirming the assay’s performance. The skin corrosion test identified *A. calamus* rhizome oil as non-corrosive under the test conditions. Tissue viability remained well above the critical threshold of 50% for both short (3 min) and prolonged (60 min) exposure times. Negligible NS_MTT_ values further confirmed the reliability of the viability readings. The individual tissue replicates’ viability for negative, positive, and *A. calamus*-treated rhizome oil treated with 3 min of exposure and 60 min of exposure is provided in Figures S7 and S8.

### 3.5. A. calamus Rhizome Oil Is Assigned to GHS Category 2, i.e., Skin Irritant

Since the skin corrosion test showed that *A. calamus* rhizome oil was non-corrosive, and the skin irritation test showed that *A. calamus* rhizome oil was an irritant, *A. calamus* rhizome oil is assigned to a GHS Category 2, i.e., skin irritant.

As the *A. calamus* rhizome oil was not corrosive, testing for skin sensitisation was performed to address the (1) molecular interaction with skin proteins, (2) inflammatory responses in keratinocytes, and (3) activation of dendritic cells. This method provides information about only one mechanistic event. A combination of the methods needs to be used in the DA to assign the skin sensitisation hazard potential.

### 3.6. A. calamus Rhizome Oil Showed Moderate Reactivity in the DPRA

The DPRA test was performed following the cysteine 1:10/lysine 1:50 prediction model (Table S1). A linear standard curve was obtained for both cysteine and lysine peptides (R^2^ > 0.99, Figure S9). All reference controls and peptide stability checks met the OECD acceptance criteria (Table 4). A mean peptide depletion threshold of 6.38% was used to discriminate sensitisers from non-sensitisers. *A. calamus* rhizome oil produced a mean cysteine peptide depletion of 46%, indicating moderate reactivity, while lysine peptide depletion was <1% (Table 5). The positive control, cinnamaldehyde, showed the expected high reactivity (81% cysteine, 67% lysine), confirming assay validity. Based on the combined mean depletion (23%), the test item was classified as a moderate sensitiser under the DPRA prediction model due to selective cysteine reactivity.

### 3.7. The KeratinoSens™ Assay Identified A. calamus Rhizome Oil as a Sensitiser

**Figure 2:**
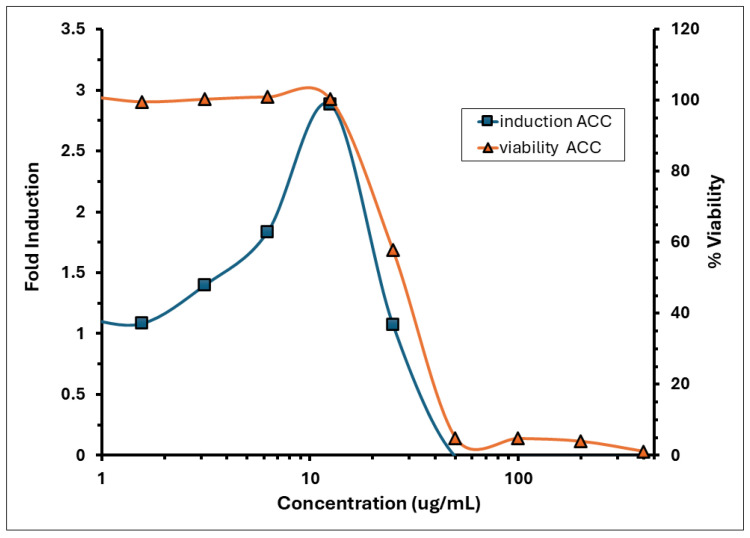
Induction and viability of *A. calamus* rhizome oil in the KeratinoSens™ assay.

The KeratinoSens™ assay was conducted to evaluate the skin sensitisation potential of the *A. calamus* rhizome oil. The results are summarised in Table 6. Key parameters, including IC_50_, I_max_, and EC_1.5_, were calculated to assess luciferase gene expression associated with the Keap1-Nrf2-ARE pathway. The IC_50_, I_max_, and EC_1.5_ for *A. calamus* rhizome oil and trans-cinnamaldehyde are presented in Table 6. The IC_50_ value, representing the concentration of the *A. calamus* rhizome oil causing 50% cell viability, was determined to be 28.51 µg/mL, and the observed IC_30_ was 21.48 µg/mL. As shown in Figure 2, cell viability remained above 75% at concentrations ≤25 µg/mL but dropped sharply at concentrations ≥50 µg/mL, indicating significant cytotoxicity. Concentration wise luciferase induction activity and cell viability is provided in Table S2.

**Figure 3:**
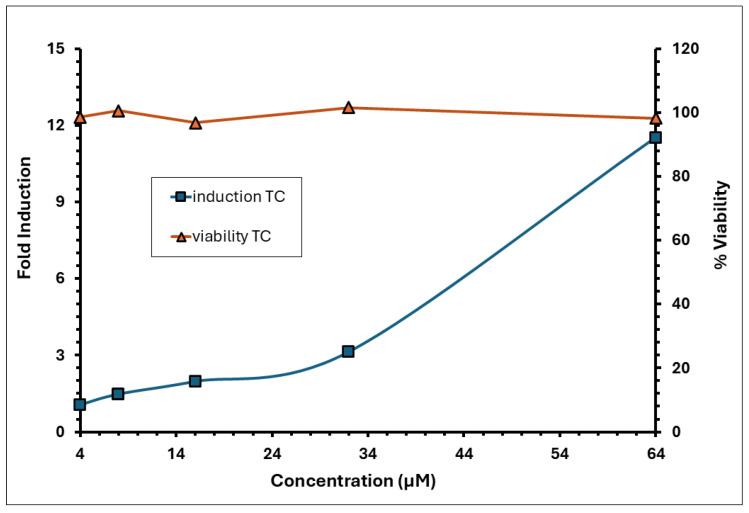
Induction and viability of trans-cinnamaldehyde (positive control–TC) in the KeratinoSens™ assay.

The induction of luciferase activity was concentration-dependent, with the maximum induction (I_max_) observed at 12.5 µg/mL, averaging 2.88 across replicates. The concentration required to achieve a 1.5-fold increase in luciferase activity (EC_1.5_) was 3.84 µg/mL. The average fold induction of luciferase activity (I_max_) for the test item exceeded 1.5-fold, reaching 1.86 and 3.17 at concentrations of 6.25 and 12.5 µg/mL, respectively, in experiment 1; and 1.82 and 2.60 at the same concentrations in experiment 2 (Figure 2). A decline in induction and an increase in cytotoxicity was observed from >12.5 µg/mL. The % CV observed for the negative control (DMSO) during experiments 1 and 2 was 11.09% and 14.03%, respectively, which was below 20%. The positive control demonstrated strong luciferase induction, with an I_max_ of 11.52 (Figure 3). The EC_1.5_ value was calculated as 8.74 µg/mL, confirming that the assay was performing correctly and confirming the positive control as a strong sensitiser.

The KeratinoSens™ assay identified *A. calamus* rhizome oil as a sensitiser, characterised by moderate cytotoxicity (IC_50_ = 28.5 µg/mL) and luciferase induction (I_max_ = 2.88, EC_1.5_ = 3.84 µg/mL).

### 3.8. A. calamus Rhizome Oil Met the Positive Response Criteria of RFI ≥ 150% for CD86 and RFI ≥ 200% for CD54, Indicating Its Sensitising Potential

**Figure 4:**
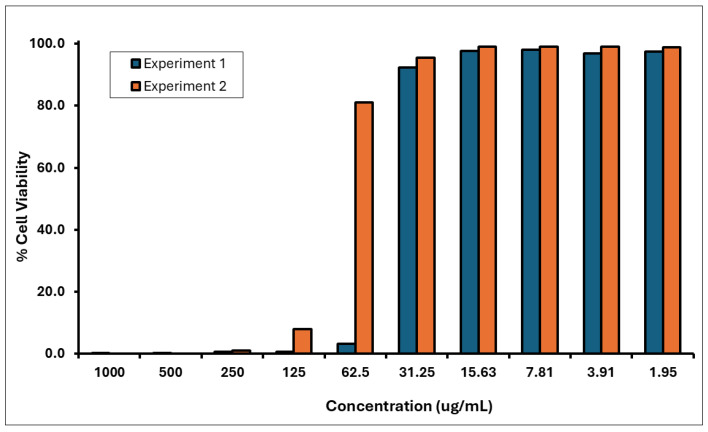
Cell viability of THP-1 Cells treated with *A. calamus* rhizome oil from concentrations between 1.95 and 1000 µg/mL.

Two independent dose range-finding assays were conducted to determine the CV_75_ value (i.e., the concentration resulting in 75% cell viability) of the *A. calamus* rhizome oil. This value was subsequently used to calculate doses for the CD86 and CD54 expression measurement experiments. Cytotoxicity (cell viability < 75%) was observed at test concentrations between 62.5 and 1000 µg/mL in DRF 1 and 125–1000 µg/mL in DRF 2 (Figure 4). The CD86 and CD54 expression measurements were performed using the CV_75_ value of 51 µg/mL to determine the RFI. Based on the CV_75_ values, the CD86/CD54 expression measurement assays were performed with concentrations ranging from 17 to 61 µg/mL.

**Figure 5:**
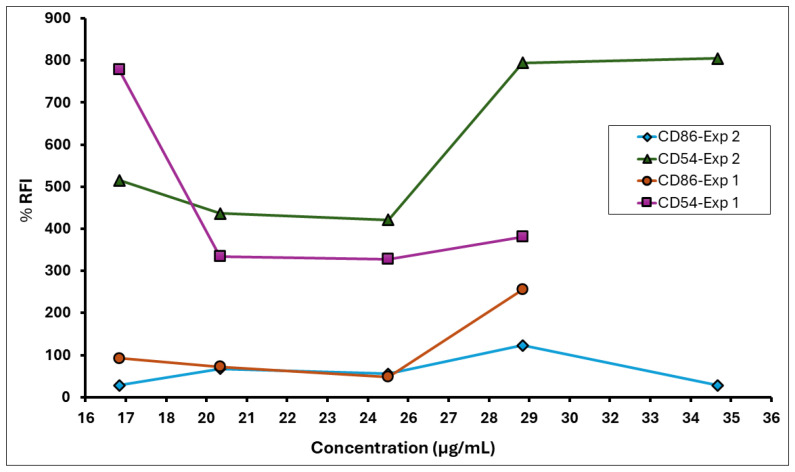
% RFI for CD86 and CD54 expression on THP-1 cells following exposure to *A. calamus* rhizome oil in the hCLAT assay.

The medium, solvent, and positive control met the assay acceptance criteria for a valid test in both experiments 1 and 2 (Table 7). The *A. calamus* rhizome oil met the positive response criteria of RFI ≥ 150% for CD86 and RFI ≥ 200% for CD54, indicating a sensitising potential (Figure 5). The calculated EC_150_ was 27 µg/mL, while the EC_200_ values could not be calculated as the RFI values for CD54 did not show a dose-dependent increase; however, all values exceeded the positive criteria. Here, we used 17 µg/mL as a surrogate minimum induction threshold (MIT) value. Results of the hCLAT assay are summarised in Table 8.

### 3.9. A. calamus Rhizome Oil Was Predicted to Be a GHS Category 1B Sensitiser

Based on the “2-out-of-3” criterion outlined in OECD TG No. 497, the *A. calamus* rhizome oil was predicted to be a skin sensitiser. The “2-out-of-3” DA can be used to make a prediction as to whether the substance is a skin sensitiser (Category 1) or not; however, it does not provide information on the skin sensitisation potency (Sub-category 1A versus 1B). Therefore, if the substance is predicted to be a skin sensitiser based on the “2-out-of-3” DA, for REACH information requirements, further information needs to be generated to conclude the skin sensitisation potency. Subsequently, the KE3/1 Sequential Testing Strategy [48,49], endorsed by the US EPA [50,51], was also applied. This approach addresses KE1 and KE3 in the AOP for skin sensitisation via the DPRA and h-CLAT, respectively, and provides both hazard identification and GHS potency classification (1A, 1B, or Not Classified). A test item with an h-CLAT minimum induction threshold (MIT) of ≤10 µg/mL is classified as “Strong” (GHS 1A), whereas an MIT of >10 µg/mL indicates “Weak” (GHS 1B), where MIT indicates the lowest value eliciting a positive outcome. The DASS app utilises the DA to predict skin sensitisation hazards, classifying substances as sensitisers or non-sensitisers and their potency based on UN GHS categories. These predictions integrate data from in vitro assays representing key events in the skin sensitisation adverse outcome pathway, along with in silico hazard assessments [52]. Using coupled data from the DPRA (mean %Cys and %Lys depletion of 23%) and hCLAT (using EC150 of 27 µg/mL), using the DASS app, *A. calamus* rhizome oil was predicted to be a GHS Category 1B sensitiser. The CD54 RFI exceeded the 200% threshold across the tested range without a clear dose response. We therefore avoided using EC200 as the primary potency anchor and used finite EC150 as MIT. While the KE 3/1 DA provides a biologically meaningful categorical potency for hazard classification, quantitative risk assessment is better anchored to a probabilistic point-of-departure (PoD) from SARA-ICE; accordingly, we report the KE3/1 Category 1B as the hazard/potency classification and use the SARA-ICE ED_01_ (subsequently combined with QRA2 exposure/SAF workflows) to derive product-use concentration limits.

Using the Scientific Committee on Consumer Safety (SCCS) default usage parameters (daily amount, frequency, and treated skin surface area), data were calculated as applied mass per application (M) and derived product-specific conservative safe concentrations (C_max_) from the SARA-ICE ED_01_ values [53,54]. The results from the SARA-ICE model are provided in Table 9. The SCCS-derived per application applied masses used here were as follows: face cream: 1.27 mg/cm^2^ (derived from daily amount of product category that is applied/received (qx) = 1.54 g/day, frequency = 2.14 use/day, and face SSA = 565 cm^2^); body lotion: 0.219 mg/cm^2^ (qx = 7.82 g/day, frequency = 2.28, and SSA = 15,670 cm^2^); hand cream: 1.26 mg/cm^2^ (qx = 2.16 g/day, frequency = 2, and SSA = 860 cm^2^); shower gel (rinse-off whole body): 0.0076 mg/cm^2^ (qx = 0.19 g/day, frequency = 1.43, and SSA = 17,500 cm^2^). Applying the point-of-departure (PoD) and QRA2 product SAFs (SAF = 100 for leave-on face/body/hand; SAF = 300 for rinse-off), the calculated provisional maxima are as follows: face cream C_max_ = 0.13% (*w*/*w*), 1300 ppm, body lotion C_max_ = 0.78%, (*w*/*w*), 7800 ppm, hand cream C_max_ = 0.13%, (*w*/*w*), 1300 ppm, and shower gel (rinse-off) C_max_ = 7.46%, (*w*/*w*) 74,600 ppm using the formula given below

$$Cmax\;\left(\%w/w\right)=\frac{PoD}{10\times M\times SAF}$$

where % C_max_ is the Provisional Maxima, PoD is the geometric mean of the ED_01_ predicated on being a sensitiser, SAF is the sensitisation assessment factor, and M is the applied mass/cm^2^. The amount applied mass/unit area (M, mg/cm^2^) was computed as (qx/f × 1000)/SSA where qx is the SCCS effective daily amount for each product category(g/day), f is the application frequency (uses/day), and SSA is the treated skin surface area (SSA, cm^2^).

The calculated safe maximum concentrations (C_max_) expressed as % (*w*/*w*) for representative consumer product categories using SCCS default applied-mass inputs are provided in Table 10.

## 4. Discussion

The use of NAMs to identify chemical hazards has accelerated in recent years [55,56]. In the crop protection industry, a battery of in vitro NAMs was used to identify the skin irritation, skin sensitisation, ocular irritation and lung irritation hazards for captan and folpet; the in vitro NAM data correlated well with existing in vivo data except for an under prediction for ocular irritation [57].

In the current DPRA, selective modification of cysteine over lysine indicates that the reactive species in the *A. calamus* rhizome oil could behave as soft Michael acceptors that favour nucleophilic thiolate attacks [58]. β-asarone, an allylbenzene in the oil’s composition, possesses an α, β-unsaturated propenyl side chain capable of 1,4-conjugate addition, a reaction mechanism entirely consistent with the observed thiol specificity [59,60]. Such cysteine-biassed depletion profiles are recurrent among botanical sensitisers of moderate human potency, lending mechanistic plausibility to the classification [61,62,63].

For the KeratinoSens™ assay, mechanistically, the modest yet clear induction pattern suggested the electrophilic modification of Keap1 cysteines by constituents present in the oil, liberating Nrf2 to translocate and drive ARE transcription. The sharp fall-off in signal at cytotoxic doses further supports that luciferase increases stem from pathway activation rather than generalised stress, since induction collapses once viability drops below 60%. For the hCLAT assay, the requirement that both CD86 and CD54 exceed their respective cut-off points at sub-toxic concentrations demonstrates that the oil can trigger the maturation signals necessary for T-cell priming [64,65]. The convergent activation of these two markers at the same concentration is a characteristic of genuine skin sensitisers, where CD54 alone may rise secondary to reactive oxygen species [66].

For the skin irritation test, the *A. calamus* rhizome oil decreased tissue viability to a mean of 23.5% (SD = 1.5%), far beneath the 50% cut-off that operationally defines an irritant response in this model. Because viability remained well above the <5% range that typifies corrosive destruction, the result is diagnostic of reversible irritation rather than irreversible corrosion. The viability loss also aligns with previous findings for essential-oil irritants whose primary constituents disrupt intercellular lipid lamellae without penetrating to the basal layer [67,68]. The chemical profile of the oil, such as β-asarone and allied phenylpropenoids, possesses surfactant-like amphiphilicity and can fluidise the *stratum corneum* lipids, precipitating local cytokine release and erythema but not the protein denaturation characteristic of strong bases or oxidisers [69,70,71]. The short recovery interval in the skin irritation test does not permit visual scoring of erythema; nevertheless, the substantial decrease in viability might be due to mitochondrial compromise sufficient to predict clinical redness and oedema [72,73,74].

The high viability of tissue treated with *A. calamus* rhizome oil in the skin corrosion test suggests a lack of irreversible protein-denaturing effects, with any membrane disruption being transient. This aligns with the amphiphilic nature of its major phenylpropenoids, which lack the extreme pK or redox potential typical of corrosives [75,76]. Despite its limitations, the RhE model’s >90% predictive accuracy supports the non-corrosive classification [77,78].

The present investigation utilised the OECD “3-Key-Event” battery, KE 3/1 Sequential Testing Strategy and the RhE irritation and corrosion assays to define the dermal hazard profile of *A. calamus* rhizome oil. For skin sensitisation, the data revealed a coherent mechanistic progression from selective cysteine conjugation in the DPRA, through Nrf2-ARE activation in keratinocytes, to the upregulation of co-stimulatory markers on THP-1 dendritic surrogates. Using the OECD TG No. 497 DA for skin sensitisation, *A. calamus* rhizome oil is defined as a skin sensitiser. Additionally, based on the KE 3/1 Sequential Testing Strategy, the rhizome oil is further categorised as a Category 1B sensitiser, i.e., moderate potency. The five-assay battery delivers a strong narrative that *A. calamus* rhizome oil fulfils every mechanistic checkpoint of the skin sensitisation AOP while remaining non-corrosive yet clearly irritant. Additionally, *A. calamus* rhizomes contain a diverse array of chemical constituents in addition to phenylpropanoids, monoterpenes, and sesquiterpenes, each contributing to the oil’s biological and dermal effects [79,80,81,82]. While its bioactive compounds contribute to its therapeutic efficacy, they also raise concerns regarding dermal toxicity. These findings align with previous studies highlighting the allergenic and irritant properties of plant-derived essential oils [83,84,85]. For example, clove oil, dominated by eugenol, shares similar electrophilic properties and sassafras oil, rich in safrole, demonstrates comparable irritation potential but poses additional systemic toxicity risks, including carcinogenicity [86,87]. Isoeugenol’s skin sensitisation potential could be due to hydroxy quinone methide formation, while quinone methide and an ortho-quinone mediate eugenol’s sensitisation potential [88]. Similarly, the skin sensitisation potential of eugenol and isoeugenol was also evaluated [89]. Enhanced luciferase induction was found in the presence of Aroclor-induced rat liver S9 in the KeratinoSens™ reporter assay for methyl-isoeugenol and eugenol. However, methyl-isoeugenol gave a weak (I_max_ 2.4) and eugenol gave no gene induction (I_max_ 1.7) in the absence of S9, suggesting their pro-hapten nature [90]. α- and β-Pinene were found to be positive (i.e., sensitiser) in the GPMT at a concentration of 4% [91] while in the LLNA, they both gave a weak response [92]. Eucalyptus and camphor oils are primarily irritant due to 1,8-cineole and camphor which act through physical disruption of the skin barrier rather than chemical sensitisation [93]. Oils like lavender and rosemary, rich in linalool and carnosic acid, respectively, demonstrate protective effects due to their antioxidant and anti-inflammatory properties [94]. Phenylpropanoids present in the oils may exacerbate irritation by inducing oxidative stress in keratinocytes, driving the inflammatory response [95]. At the concentrations tested in the skin irritation test, the oils’ components induce sufficient inflammatory responses to qualify as irritants without causing the extensive tissue damage associated with corrosion. Corrosive agents typically cause full-thickness necrosis by penetrating beyond the epidermis to the dermis, denaturing proteins and destroying cellular membranes [96]. Some minor constituents, such as linalool and caryophyllene, in the oil might provide partial anti-inflammatory and antioxidant effects, mitigating the overall dermal reactivity of the oil [97,98]. The results emphasise the importance of using *A. calamus* rhizome oil in appropriately diluted concentrations and formulating it with agents that may mitigate its irritant effects and the need for regulatory oversight. The concentration of compounds, along with the frequency, duration of exposure, and skin condition, could be considered important factors. The SARA-ICE DA Model predicted an ED_01_ (50th percentile) of 180 µg/cm^2^, and a range spanning from 2.0 µg/cm^2^ (5th percentile) to 13,000 µg/cm^2^ (95th percentile) with PoD being 170 µg/cm^2^.

The probabilistic ED_01_ distribution already accounts for inter-individual and model variability in human dose–response data. The QRA2 SAF, in contrast, aggregates additional uncertainties not captured by potency estimations, namely (1) matrix and formulation effects (vehicle influence, volatility, and occlusion); (2) exposure modelling variability (frequency, surface area, and product amount); (3) inter- and intra-individual physiological variability; and (4) database and read-across limitations. By choosing the OECD DA PoD + SAF as the primary scenario, we separate statistical uncertainty (captured in PoD derivation) from extrapolation uncertainty (captured in SAF). This layered yet non-redundant strategy aligns with contemporary risk-assessment principles [99,100].

Furthermore, the SCCS-based applied masses show that consumer leave-on facial and hand creams deliver 1.25 mg/cm^2^ per application, for example, producing face cream C_max_ of 0.13% (*w*/*w*). On the other hand, rinse-off products distribute small, retained masses over large surface areas, yielding much higher permissible concentrations. For example, the shower gel C_max_ is 7.46% (*w*/*w*), but rinse-off classification also implies different retention and absorption dynamics, so interpretation must be cautious. The higher permissible levels for rinse-off products reflect transient skin contact and limited retention; however, final concentrations should also consider dermal absorption kinetics and potential cumulative exposure across products. Where aggregate exposure is relevant, the per-product C_max_ must be adjusted downward or some categories removed to achieve an acceptable margin.

The increasing regulatory acceptance of DAs and NAM batteries makes the present integration of DPRA, KeratinoSens™, and h-CLAT with SARA-ICE potency modelling particularly timely for cosmetics safety assessments. OECD TG No. 497 formally recognises that DAs can be used to replace animal tests for hazard and potency decision-making when implemented with a fixed data interpretation procedure, and the SARA-ICE workflow is explicitly described in the supporting OECD materials as an implementation route for deriving ED_01_-based PoDs. Historically, the fragrance industry established the QRA concept and QRA2 refined it into a transparent framework (NESIL to AEL and to CEL) with composite sensitisation assessment factors (SAFs) that can be used to translate pos probabilistic or Bayesian PoD derivation, continuous PoDs, percentile selection [101], as well as practical NGRA case studies, and weight of evidence approach methods for skin sensitisation potency categorisation [102], which supports that NAM data can be combined with SAF-based extrapolation to produce both a recommended regulatory limit and precautionary sensitivity bounds for exposure modelling to fragrance and botanical constituents for vulnerable subpopulations. With this said, applying this approach to botanicals requires additional safeguards. Natural oils are chemically complex and chemotypes can vary geographically and by extraction method; consequently, compositional standardisation, specification of analytical marker content (for instance β-asarone in *A. calamus*), and batch control are prerequisites before a single NAM-derived PoD can be reliably applied across commercial supplies. Taken together, the regulatory potential of the NAM-anchored, DA-driven potency estimates can be integrated into the QRA2 workflow to yield product-specific safe concentrations, but only when supported by robust exposure characterisation, compositional specification, and, where possible, targeted empirical data (dermal absorption or limited human tolerance studies) to reduce residual uncertainty while emphasising that its defensible application to botanicals rests on compositional control. Robust exposure characterisation (or conservative SCCS defaults) and transparent presentation of both primary (OECD-DA PoD + QRA2 SAF) and precautionary scenarios can also provide regulators with transparent options that balance precaution with practicability and align with current NGRA best practice and the evolving SCCS/OECD expectations for cosmetic safety assessments.

## 5. Conclusions

This study delivers a comprehensive in vitro toxicological evaluation of *A. calamus* rhizome oil, leveraging a full suite of OECD-compliant NAMs. The oil was classified as a GHS Category 1B skin sensitiser and a Category 2 irritant, while demonstrating a non-corrosive profile. These findings were supported by mechanistic evidence across all three key events of the skin sensitisation AOP: protein reactivity (DPRA), keratinocyte activation (KeratinoSens™), and dendritic cell activation (h-CLAT). The study highlights the utility of NAMs to replace traditional animal-based assays with mechanistically informed, regulatory-accepted tests. This approach not only improves ethical and scientific standards but also enables hazard identification and potency categorisation. Chemical analysis revealed high β-asarone content (40.75%), reinforcing the need for compositional control due to its known toxicological risks. Risk modelling using the SARA-ICE Model and SCCS parameters established conservative safe concentration limits for topical applications, ranging from 0.13% to 0.78% (*w*/*w*) for leave-on products and up to 7.46% (*w*/*w*) for rinse-off formulations. These data fill a critical gap in the safety assessment of *A. calamus* oil and support its regulated use in consumer products. While the study integrates NAM-based in vitro and potency modelling, a few limitations should be acknowledged. The probabilistic point of departure values are model-derived and not yet supported by human patch-test validation; dermal absorption kinetics were assumed rather than measured; and the compositional variability inherent to natural oils may influence sensitisation potency. These uncertainties, although mitigated through structured assessment factors (SAFs), highlight the need for future empirical work on human exposure, bioavailability, and formulation effects. Overall, this work exemplifies the application of NAMs in botanical safety evaluation and provides a robust framework for future assessments of complex natural ingredients. This study also forms a base to demonstrate how NAM-derived probabilistic potency data (SARA-ICE) can be integrated quantitatively with SCCS QRA2 frameworks, enabling reproducible, evidence-based safety limits for complex natural mixtures. Continued research should focus on formulation-level testing, chemotype standardisation, and strategies to mitigate dermal reactivity.

## Figures and Tables

**Table 1:** Quantification of α-, β-, and γ-asarone in the *A. calamus* rhizome oil.

Asarone	Retention Time (Reference)	Retention Time (Sample)	Reference Standard Peak Area	Sample Peak Area	% Content
α	6.402	6.452	8,178,131	34,024	4.16
β	5.627	5.670	2,252,390	1,147,401	40.75
γ	5.988	ND	2236	ND	ND

ND: not detected.

**Table 2:** Viability and non-specific MTT reduction (NS_MTT_) for *A. calamus* rhizome oil in the skin irritation test.

Sample Type	Mean % Viability	SD	CV (%)	NS_MTT_ (%)
DPBS (Negative Control)	100	0.037	1.91	-
*A. calamus* Rhizome Oil	23.5	1.50	6.39	−11.2
5% SDS (Positive Control)	1.5	0.058	3.87	-

**Table 3:** Viability and non-specific MTT reduction (NS_MTT_) for *A. calamus* rhizome oil in the skin corrosion test.

Exposure Time (min)	Sample Type	Mean % Viability	SD	CV (%)	NS_MTT_ (%)
3	Distilled Water (Negative Control)	100	0.04	1.87	N/A
	*A. calamus* Rhizome Oil	92.72	2.13	2.30	−0.7
60	Distilled Water (Negative Control)	100	0.04	1.88	N/A
	*A. calamus* Rhizome Oil	91.17	1.49	1.63	0.1
	8N Potassium Hydroxide (Positive Control)	0.17	0.03	17.65	−18.6

N/A = not applicable.

**Table 4:** Acceptance criteria and observed results for the DPRA using cinnamic aldehyde as the positive control. All parameters met OECD TG No. 442C requirements, confirming assay validity.

Acceptance Criteria	Obtained Results
The standard calibration curve should have an R^2^ > 0.99.	>0.99
The mean % peptide depletion value of the three replicates for the positive control cinnamic aldehyde should be between 60.8% and 100% for the cysteine peptide.	81%
The mean % peptide depletion value of the three replicates for the positive control cinnamic aldehyde should be between 40.2% and 69.0% for the lysine peptide.	67%
The maximum standard deviation (SD) for the positive control replicates should be <14.9% for cysteine depletion and <11.6% for lysine depletion.	0.29% (Cys)/0.37% (Lys)
The mean peptide concentration of reference control A should be 0.50 ± 0.05 mM.	0.5–0.51 mM
The coefficient of variation (CV) of peptide peak areas for the nine reference controls, B and C in acetonitrile should be <15.0%.	0.38% (Ref B)/1.65% (Ref C)

**Table 5:** Cysteine and lysine depletion for *A. calamus* rhizome oil in the DPRA.

**Peptide**	**Sample**	**% Depletion**		**%Mean Peptide Depletion** **(Cysteine and Lysine Peptide)**	
				***A. calamus*** **Rhizome Oil**	**Cinnamaldehyde**
		**Mean**	**SD**	23	74
Cysteine	*A. calamus* Rhizome Oil	46	0.52		
	Cinnamaldehyde	80.99	0.29		
Lysine	*A. calamus* Rhizome Oil	0.47	0.18		
	Cinnamaldehyde	67	0.37		

**Table 6:** Cytotoxicity (IC_50_) and luciferase induction (I_max_ and EC_1.5_) for *A. calamus* rhizome oil and trans-cinnamaldehyde (positive control) in the KeratinoSens™ assay.

Sample	IC_30_	IC_50_	I_max_	EC_1.5_
*A. calamus* Rhizome Oil	21.48	28.51	2.88 ± 0.40	3.84 ± 0.21 *
Trans-cinnamaldehyde	N/A	N/A	11.52 ± 10.14	8.84 ± 1.86 µM

N/A = not applicable, * = values are in µg/mL; all values are represented as mean ± SD.

**Table 7:** Summary of CD86/CD54 expression, cell viability, and relative fluorescence intensity (RFI) in h-CLAT assay controls across two experiments, demonstrating assay validity and sensitisation thresholds.

	MFI Ratios (CD86/CD54)			% Viability			% RFI (CD86/CD54)	
Controls	Experiment 1	Experiment 2	Criterion	Experiment 1	Experiment 2	Criterion	Experiment 1	Experiment 2
Medium Control	131.33/ 111.45	122.56/ 115.24	>105	96.50	97.90	>90	N/A	
Solvent (DMSO) Control	149.04/ 120.38	124.29/ 110.73		95.10	96.70		148.08/ 168.42	116.22/ 76
Positive Control	N/A	N/A		87.70	67.10	>50	476.62/ 1140.63	537.21/ 1342.11

N/A = not applicable.

**Table 8:** Summary of the human Cell Line Activation Test (h-CLAT) results for *A. calamus* rhizome oil, including individual values for CD86 and CD54 induction in experiments 1 and 2.

CV_75_ Value (µg/mL)	Experiment 1	Experiment 2	EC_150_ (µg/mL)	* EC_200_ (µg/mL)	Final Prediction
51	P_12_	P_2_	27	17	Positive

* = Although positive, the EC200 and MIT could not be calculated. The lowest dose tested was 17 µg/mL/mL (which yielded a positive response) and was used as a surrogate value. P_12_ = positive for both markers, P_2_ = positive for the CD54 marker only.

**Table 9:** SARA-ICE model results.

PoD	ED_01_ 5th	ED_01_ 50th	ED_01_ 95th
170	2.0	180	13,000

**Table 10:** Calculated safe maximum concentrations (C_max_) expressed as % (*w*/*w*) (and ppm in parentheses) for representative consumer product categories using SCCS default applied-mass inputs using the OECD SARA-ICE DA PoD (170 µg/cm^2^).

Product Category (SCCS Mapping)	SCCS-Derived Applied Mass per Application, M (mg/cm^2^)	SAF (QRA2)	C_max_ (%, *w*/*w*) [ppm]
Face cream (leave-on)	1.27	100	0.13% [1300 ppm]
Body lotion (leave-on, whole body)	0.219	100	0.78% [7800 ppm]
Hand cream (leave-on)	1.26	100	0.13% [1300 ppm]
Shower gel (rinse-off, whole body)	0.0076	300	7.46% [74,600 ppm]

Notes: Applied mass (M) values were derived from SCCS default use parameters (daily amount, frequency, and skin surface area) following SCCS Notes of Guidance procedures. The per-application M values shown are the SCCS-derived mass of product deposited per cm^2^ of treated skin at a single use [53]; SAF = overall sensitisation assessment factor from QRA2 mapped to product category (leave-on = 100; rinse-off whole-body = 300). If an alternative SAF is justified (e.g., hair mapping, SAF = 30), corresponding C_max_ values will differ and should be reported; rounding: percentages are shown to two significant digits appropriate for reporting; ppm shown in parentheses for clarity (1% = 10,000 ppm); these C_max_ values are provisional and should be interpreted in the context of aggregate exposure (multiple products), formulation-specific dermal absorption, and weight-of-evidence integration with orthogonal NAM/human data.

## Data Availability

The original contributions presented in this study are included in the article/Supplementary Materials. Further inquiries can be directed to the corresponding authors.

## Supplementary Materials

The following supporting information can be downloaded at https://www.mdpi.com/article/10.3390/toxics13121006/s1: Figure S1: *Acorus calamus* rhizome (Left) and *Acorus calamus* rhizome powder (right); Figure S2: Hydro distillation by the Clevenger apparatus (left), *Acorus calamus* rhizome powder in an oil bath (middle), and extracted mixture (right); Figure S3: Reference standard solution of α-asarone; Figure S4: Reference standard solution of β-asarone; Figure S5: Reference standard solution of γ-asarone; Figure S6: Tissue viability for *A. calamus* rhizome oil (ACC) and negative controls in the skin irritation test; Figure S7: Tissue viability for *A. calamus* rhizome oil (ACC) and negative control in the skin corrosion test (3 min of exposure); Figure S8: Tissue viability for *A. calamus* rhizome oil (ACC) and controls in the skin corrosion test (60 min of exposure); Figure S9: Cysteine and lysine standard curve; Table S1: Prediction model as per OECD 442c for the DPRA prediction; Table S2: Luciferase induction activity and cell viability for *A. calamus* rhizome oil in the KeratinoSens™ assay; Text S1: Skin Irritation; Text S2: Skin Corrosion; Text S3:The Direct Peptide Reactivity Assay.
