# Supplementary material for: Comprehensive In Vitro Safety Assessment of Acorus calamus Rhizome Oil Using OECD-Compliant New Approach Methods: Classification as a GHS Category 1B Sensitiser and Category 2 Irritant
Source: Toxics. 2025 Nov 21;13(12):1006. doi: 10.3390/toxics13121006 (PMC12737251; doi:10.3390/toxics13121006)
Supplement: Supplementary file 1 [file toxics-13-01006-s001.zip › toxics-3961180-supplementary.pdf]

Test S1: **Skin Irritation**

Upon arrival, RhE tissues were meticulously cleaned of basal agarose using Kim wipes and subsequently transferred to 6-well plates filled with fresh growth medium (1 mL). The tissues underwent a pre-incubation period of at least 2 hours at a temperature of 37 ± 1°C within an incubator that maintained a 5±1% CO_2 concentration and a humidified environment. An application of A. calamus rhizome oil (16 µL/ 0.5 cm^2) was administered directly onto the tissues. Sterile DPBS served as the negative control, while sodium dodecyl sulphate (SDS, 5%) was utilized as the positive control. Each condition was replicated three times, and a nylon mesh was positioned over the tissues for uniform distribution of the test item across the tissue. Additional freeze-killed tissues were prepared to account for non-specific MTT reduction (NSMTT). After 42 minutes of exposure at room temperature, tissues were rinsed thoroughly with DPBS, dried with sterile absorbent paper, and transferred to the fresh growth medium for a 42-hour post-exposure incubation at 37 ± 1°C in a 5 ± 1% CO2 atmosphere. After the incubation period, the tissues were placed into 24-well plates that contained 0.3 mL of MTT solution (1.0 mg/mL) and incubated for 180 minutes at a temperature of 37 ± 1°C within a 5 ± 1% CO2 environment. The purple formazan salt was obtained by immersing the tissues in isopropanol (1.5 mL) for 2 hours while gently shaking and ensuring protection from light. The optical density of the extracted formazan was assessed at 570 nm using a Bio-Tek Synergy HT microplate reader (BioTek Instruments, USA).

Test S2: **Skin Corrosion**

Upon receipt, RhE tissues were cleansed of basal agarose and subsequently transferred to 6-well plates that contained fresh Maintenance Medium (1 mL). The tissues underwent pre-incubation overnight at a temperature of 37 ± 1°C within a 5 ± 1% CO2 environment. A. calamus rhizome oil (40 µL/ 0.5 cm^2) was directly applied to the tissues. Sterile distilled water served as the negative control, while potassium hydroxide (KOH, 8N) was utilized as the positive control. The tissues were subjected to exposure on the skin for durations of 3 and 60 minutes. Each condition included three replicates, with a nylon mesh applied post-application of the rhizome oil for the uniform distribution of the test item. Freeze-killed tissues were prepared to account for NSMTT. Following exposure, the tissues were rinsed ~20 times with DPBS, dried using sterile absorbent paper, and then transferred to a fresh Maintenance Medium. Finally, the tissues were transferred to 24-well plates containing MTT solution and analyzed following a procedure similar to that described for the skin irritation test above.

Test S3: The Direct Peptide Reactivity Assay

The DPRA focuses on the initial KE in the AOP related to skin sensitization, specifically determining the covalent binding of electrophilic compounds to nucleophilic amino acid residues (such as cysteine and lysine) found in proteins. The DPRA quantitatively evaluates the depletion of synthetic peptides that contain cysteine or lysine residues following exposure to the test substance. The analysis of peptide depletion is performed using HPLC, with the interpretation performed as a percentage of peptide depletion in comparison to the control samples. Chemicals are classified into sensitizing or non-sensitizing categories based on predefined thresholds for peptide reactivity. This methodology is thoroughly detailed in OECD TG 442c, DB-ALM 154, and is extensively utilized as part of the DA outlined in OECD TG No. 497 for evaluating skin sensitization potential.

Cysteine peptide (Ac-RFAACAA-COOH; MW: 750.87 g/mol) and lysine peptide (Ac-RFAAKAA-COOH; MW: 775.91 g/mol) were obtained from RS Synthesis, USA and freshly prepared immediately before each experiment. Cysteine peptide was dissolved in phosphate buffer (pH 7.5) at a final concentration of 0.667 mM. Lysine peptide was dissolved in ammonium acetate buffer (pH 10.2) at a final concentration of 0.667 mM. Cinnamaldehyde (obtained from Sigma-Aldrich) was used as a positive control at a concentration of 100 mM in acetonitrile for both cysteine and lysine peptides.

The *A. calamus* rhizome oil was weighed into sterile vials and dissolved in acetonitrile (HPLC grade) to prepare a stock solution of 20 mg/mL, which was immediately used. The cysteine peptide reaction mixture was prepared with peptide stock (750 µL), acetonitrile (200 µL), and *A. calamus* rhizome oil solution (50 µL) at a 1:10 (v/v) ratio. The lysine peptide reaction mixture was prepared with peptide stock (750 µL) and *A. calamus* rhizome oil solution (250 µL) at a 1:50 (v/v) ratio. The reaction mixtures were capped, vortexed, and incubated in the dark at 22.5-30.0°C for 24 ± 2 hours. The samples were visually inspected for precipitation, with no precipitation observed.

The analysis of peptides via HPLC was performed utilizing a Zorbax SB-C18 column (2.1 mm × 100 mm, 3.5 µm) in conjunction with a Phenomenex Security Guard C18 guard column. Detection was achieved using a fixed-wavelength UV detector calibrated to 220 nm. Mobile phase A consisted of trifluoroacetic acid (TFA) diluted in Milli-Q water (0.1%, v/v), while mobile phase B comprised TFA in acetonitrile (0.085%, v/v). The programme was set at the flow rate of 0.35 mL/min, with the total run time being 20 minutes. Prior to analysis, the system was equilibrated with mobile phase A (50%) and mobile phase B (50%) for a duration of 2 hours.

Reference Control A was a fresh peptide solution mixed with acetonitrile and analyzed immediately to verify calibration and accuracy. Reference Control B was Reference Control A after incubation for 24 ± 2 hours to assess peptide stability. The co-elution controls were the reaction mixture without peptide to ensure the *A. calamus* rhizome oil did not interfere with peptide detection. Peptide depletion was calculated based on HPLC peak areas relative to controls. The HPLC system's suitability was determined by the standard calibration curve. The

HPLC sequence was designed to ensure the first injection occurred within 24 ± 2 hours of incubation. All samples were analyzed within 30 hours.

Any negative depletion values were treated as "0" when computing the mean. According to the cysteine 1:10 / lysine 1:50 prediction model, a threshold of 6.38% average peptide depletion was used to distinguish between skin sensitizer and non-sensitizer. Additionally, applying this prediction model to classify a test item into a reactivity category of low, moderate, or high may be useful for informing potency assessments within the DA framework.

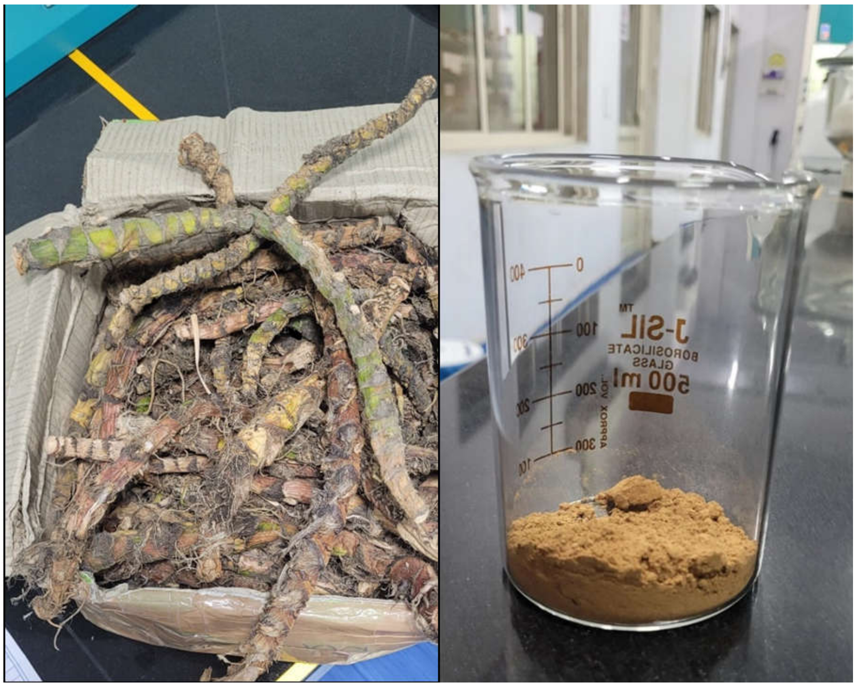

Figure S1. *Acorus calamus* rhizome (Left) and *Acorus calamus* rhizome powder (right).

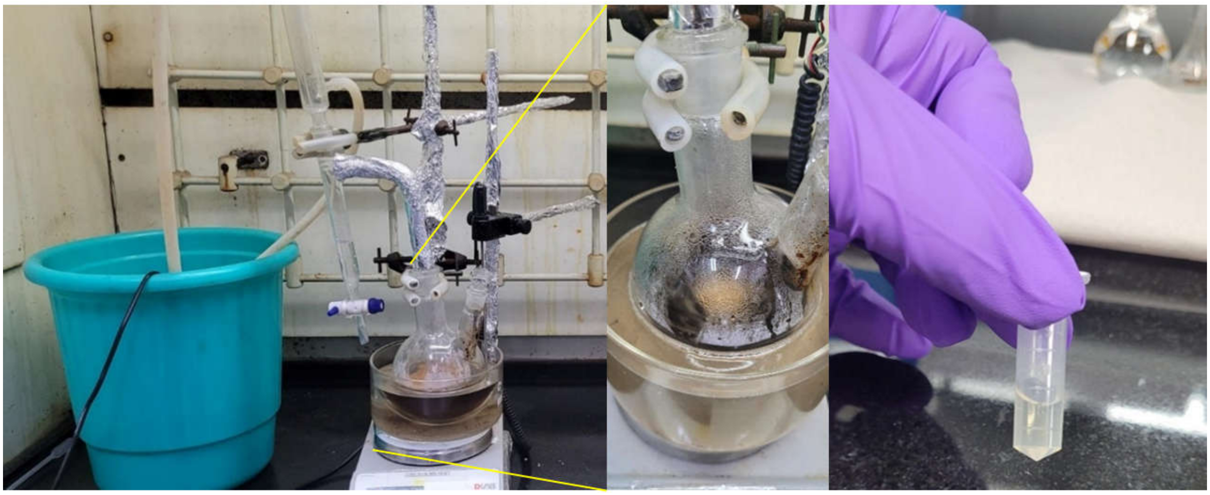

Figure S2. Hydro distillation by the Clevenger apparatus (left), *Acorus calamus* rhizome powder in an oil bath (middle), and extracted mixture (right).

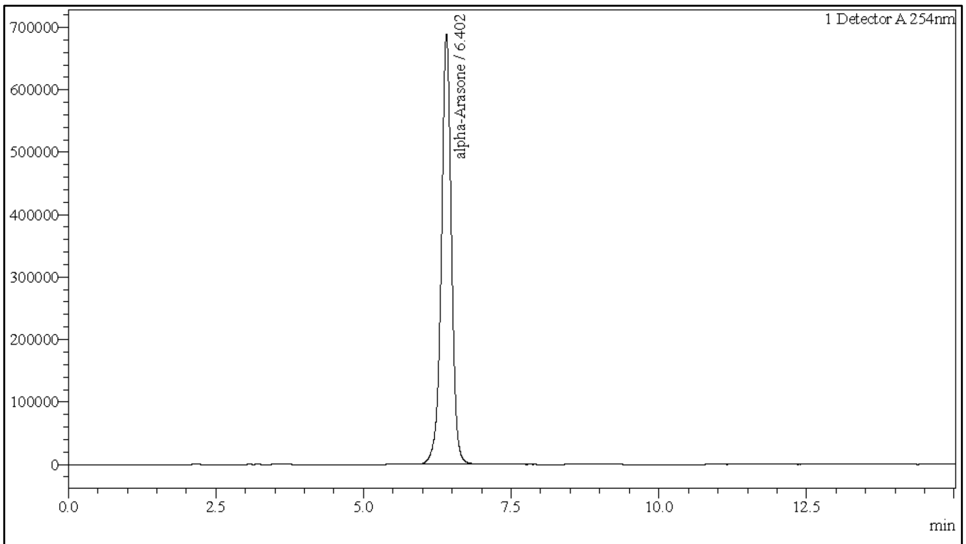

Peak Table RES_02.lcd

Detector A 254nm

Peak#	Ret. Time	Area	Height	Number of Theoretical Plate(USP)	Tailing Factor	Name
1	6.402	8178131	689647	6880	0.970	alpha-Arasone
Total		8178131	689647			

Figure S3. Reference standard solution of α-asarone.

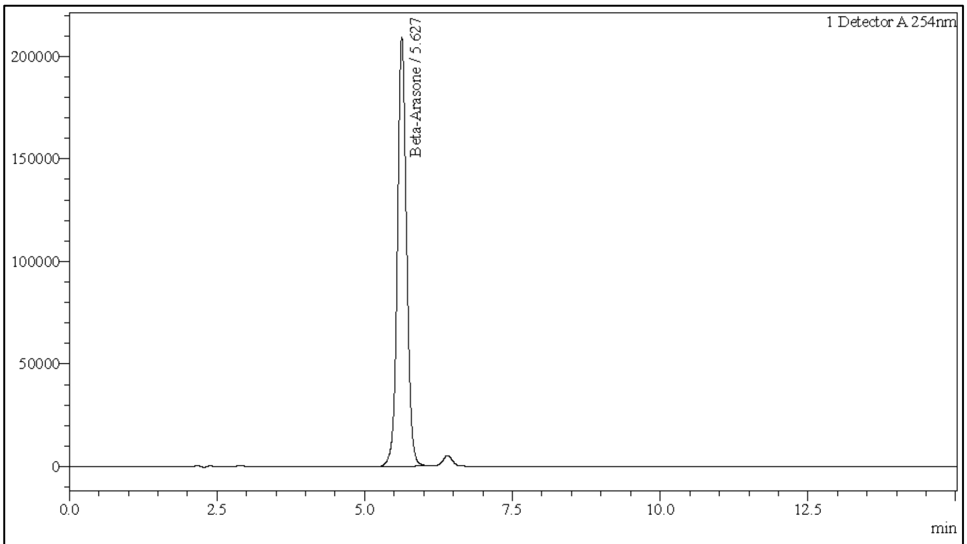

Peak Table RES_03.lcd

Detector A 254nm

Peak#	Ret. Time	Area	Height	Number of Theoretical Plate(USP)	Tailing Factor	Name
1	5.627	2252390	209332	6288	1.037	Beta-Arasone
Total		2252390	209332			

Figure S4. Reference standard solution of β-asarone.

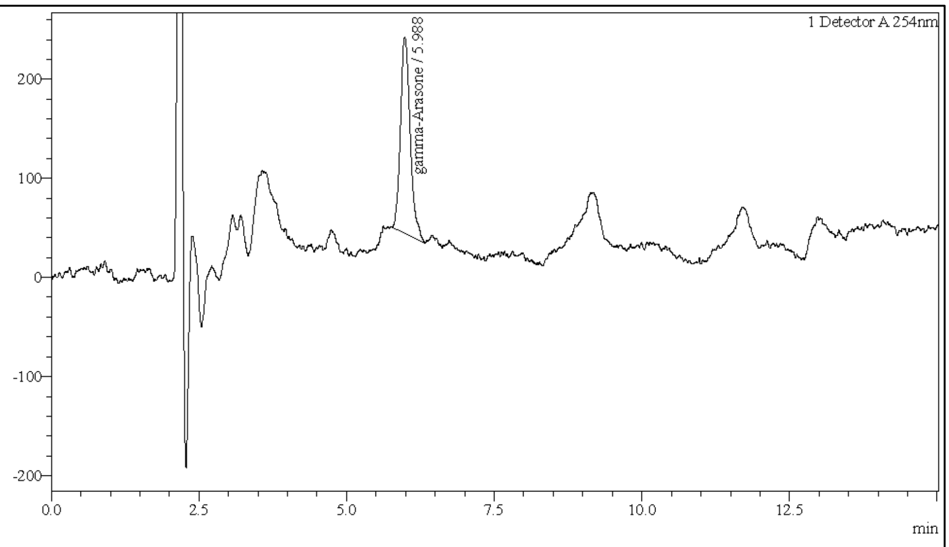

Peak Table RES_04.lcd

Detector A 254nm

Peak#	Ret. Time	Area	Height	Number of Theoretical Plate(USP)	Tailing Factor	Name
1	5.988	2236	198	6454	1.262	gamma-Arasone
Total		2236	198			

Figure S5. Reference standard solution of γ-asarone.

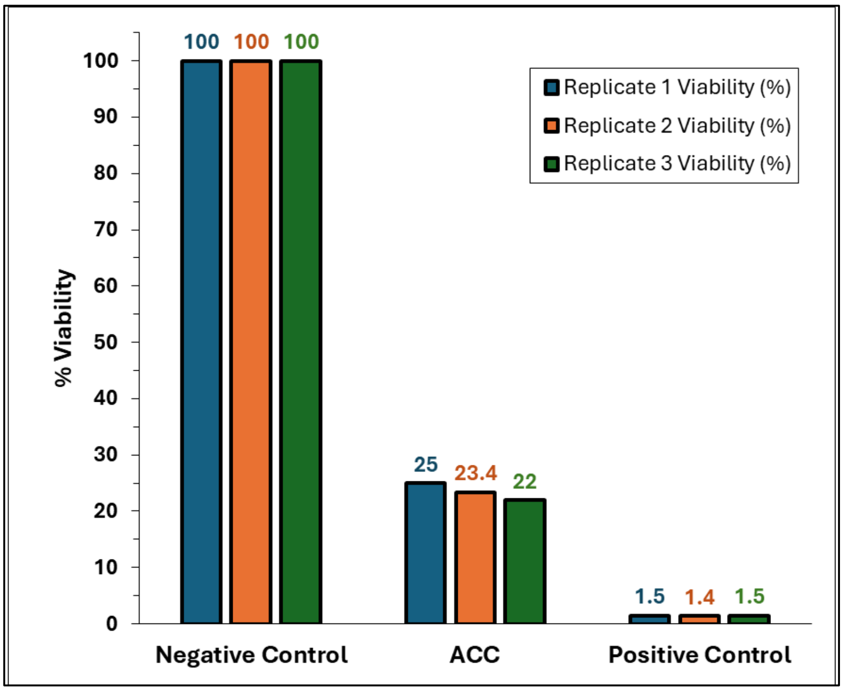

Figure S6. Tissue viability for *A. calamus* rhizome oil (ACC) and negative controls in the skin irritation test.

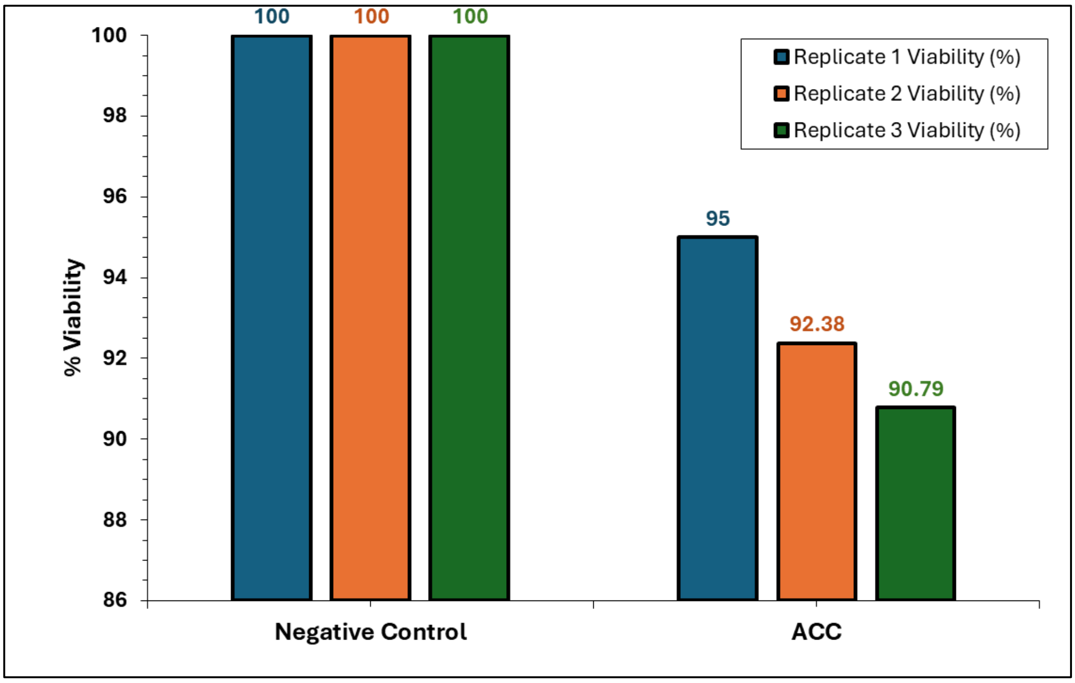

Figure S7. Tissue viability for *A. calamus* rhizome oil (ACC) and negative control in the skin corrosion test (3 minutes of exposure).

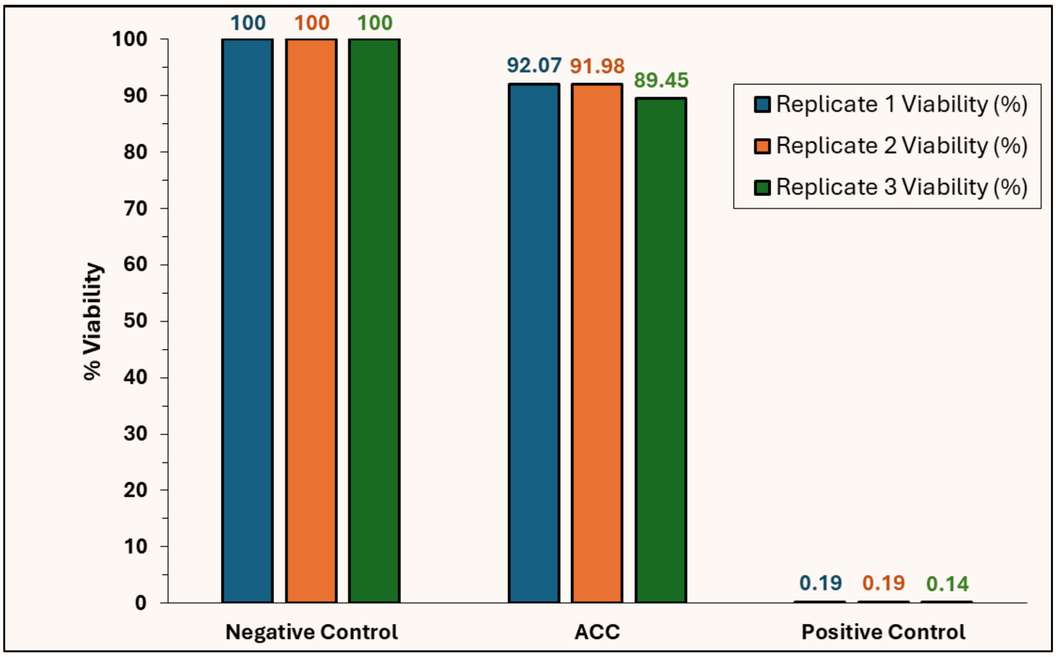

Figure S8. Tissue viability for *A. calamus* rhizome oil (ACC) and controls in the skin corrosion test (60 minutes of exposure). ACC = *A. calamus* rhizome oil.

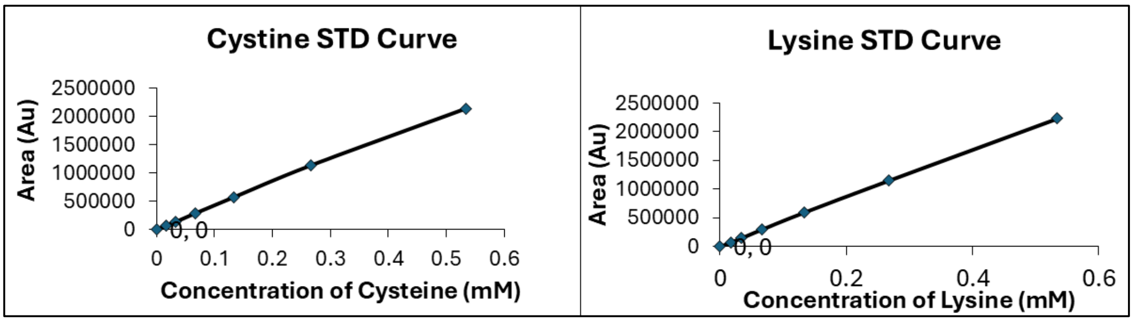

Figure S9. Cysteine and lysine standard curve.

Mean % Depletion Mean of cysteine and lysine (% Depletion)	Reactivity Class	DPRA Prediction
0% ≤ mean ≤ 6.38%	No or Minimal Reactivity	Negative
6.38% < mean ≤ 22.62%	Low Reactivity	Positive
22.62% < mean ≤ 42.47%	Moderate Reactivity	
42.47% < mean ≤ 100%	High Reactivity	

Table S1. Prediction model as per OECD 442c for the DPRA prediction.

A. calamus Rhizome Oil Concentration (µg/mL)	Mean Induction	Mean Viability (%)
0.20	0.83	99.31
0.39	1.15	99.47
0.78	1.13	101.08
1.56	1.08	99.57
3.13	1.40	100.30
6.25	1.84	100.93
12.5	2.88	100.36
25.0	1.08	57.83
50.0	-0.01	4.89
100	-0.01	4.76
200	-0.01	3.97
400	-0.01	1.12

Negative induction values at higher concentrations are attributed to cytotoxic effects due to a reduction in cell viability.

Table S2. Luciferase induction activity and cell viability for *A. calamus* rhizome oil in the KeratinoSens™ assay.
